# Size Distribution of Microparticles: A New Parameter to Predict Acute Lung Injury After Cardiac Surgery With Cardiopulmonary Bypass

**DOI:** 10.3389/fcvm.2022.893609

**Published:** 2022-04-29

**Authors:** Hao-Xiang Yuan, Kai-Feng Liang, Chao Chen, Yu-Quan Li, Xiao-Jun Liu, Ya-Ting Chen, Yu-Peng Jian, Jia-Sheng Liu, Ying-Qi Xu, Zhi-Jun Ou, Yan Li, Jing-Song Ou

**Affiliations:** ^1^Division of Cardiac Surgery, Heart Center, The First Affiliated Hospital, Sun Yat-sen University, Guangzhou, China; ^2^National-Guangdong Joint Engineering Laboratory for Diagnosis and Treatment of Vascular Diseases, Guangzhou, China; ^3^NHC key Laboratory of Assisted Circulation, Sun Yat-sen University, Guangzhou, China; ^4^Guangdong Provincial Engineering and Technology Center for Diagnosis and Treatment of Vascular Diseases, Guangzhou, China; ^5^Division of Hypertension and Vascular Diseases, Heart Center, The First Affiliated Hospital, Sun Yat-sen University, Guangzhou, China; ^6^Guangdong Provincial Key Laboratory of Brain Function and Disease, Guangzhou, China

**Keywords:** microparticles, cardiac surgery, acute lung injury, cardiopulmonary bypass, size distribution

## Abstract

**Background:**

Acute lung injury (ALI) is a common complication after cardiac surgery with cardiopulmonary bypass (CPB). No precise way, however, is currently available to predict its occurrence. We and others have demonstrated that microparticles (MPs) can induce ALI and were increased in patients with ALI. However, whether MPs can be used to predict ALI after cardiac surgery with CPB remains unknown.

**Methods:**

In this prospective study, 103 patients undergoing cardiac surgery with CPB and 53 healthy subjects were enrolled. MPs were isolated from the plasma before, 12 h after, and 3 d after surgery. The size distributions of MPs were measured by the LitesizerTM 500 Particle Analyzer. The patients were divided into two subgroups (ALI and non-ALI) according to the diagnosis of ALI. Descriptive and correlational analyzes were conducted between the size distribution of MPs and clinical data.

**Results:**

Compared to the non-ALI group, the size at peak and interquartile range (IQR) of MPs in patients with ALI were smaller, but the peak intensity of MPs is higher. Multivariate logistic regression analysis indicated that the size at peak of MPs at postoperative 12 h was an independent risk factor for ALI. The area under the curve (AUC) of peak diameter at postoperative 12 h was 0.803. The best cutoff value of peak diameter to diagnose ALI was 223.05 nm with a sensitivity of 88.0% and a negative predictive value of 94.5%. The AUC of IQR at postoperative 12 h was 0.717. The best cutoff value of IQR to diagnose ALI was 132.65 nm with a sensitivity of 88.0% and a negative predictive value of 92.5%. Combining these two parameters, the sensitivity reached 92% and the negative predictive value was 96%.

**Conclusions:**

Our findings suggested that the size distribution of MPs could be a novel biomarker to predict and exclude ALI after cardiac surgery with CPB.

## Introduction

Cardiac surgery with cardiopulmonary bypass (CPB) is a traumatic procedure and prone to postoperative acute lung injury (ALI) (1–3). The incidence of ALI after CPB surgery is about 15–60%, and the incidence of ALI in children with CPB surgery is as high as 60%. ALI-related mortality in the general population reaches 40%, and may go up to 80% in the patients after cardiac surgery with CPB, which makes ALI a major cause of increased mortality after cardiac surgery (4, 5). The mechanism by which cardiac surgery induces ALI remains unclear but may involve inflammation, activation of coagulation, increased permeability of the alveolocapillary barrier, epithelium dysfunction (6), and alveolar epithelium cell apoptosis (7). More importantly, no precise method is currently available to predict ALI after cardiac surgery.

Circulating microparticles (MPs) are a group of membrane vesicles generated from a variety of sources including endothelial cells, neutrophils, platelets, etc. upon activation or apoptosis. We and other researchers have demonstrated that circulating MPs are elevated in cardiovascular diseases including valve heart disease, acute coronary syndromes, congenital heart disease, and cardiac surgery (8–15). MPs have been proved to have important physiological and pathophysiological functions and have been suggested as a promising biomarker to diagnose and predict different stages of diseases (11, 16–26). We also demonstrated that MPs were significantly increased, impairing endothelial function and vasodilation which may cause hemodynamic instability after cardiac surgery (9, 10, 18, 27). We previously found that endothelial microparticles were increased in patients after cardiac surgery and could induce ALI (10, 17). In the early stage of ALI, alveolar macrophage-derived MPs contain activated TNF-α, which may arouse a strong effect on inflammation and immunomodulation (28). Procoagulant tissue factor-bearing MPs may lead to lung injury through activated coagulation factors X and Xa and induce pulmonary fibrosis in interstitial lung diseases (29). In the rat model of ischemia-reperfusion-induced lung injury, elevated circulating MPs carrying miR-155 increase the pulmonary vascular permeability, leading to lung injury (30). Moreover, caspase-1 contained in monocyte-derived MPs mediates the apoptosis of alveolar epithelium cells (31). Recently, we found that MPs from cardiac surgery with CPB contained many pro-inflammatory proteins such as C-reactive protein, myeloperoxidase, serum amyloid A, S100 calcium-binding protein A8, and S100 calcium-binding protein A9, which may induce severe inflammatory response and ALI (32). Indeed, the circulating angiotensin-converting enzyme-positive endothelial MPs were increased in ALI (33). However, it is unclear whether MPs can be used to predict ALI after cardiac surgery.

In this study, we measured the number and size distribution of MPs using a new technique in the patients who underwent cardiac surgery with CPB. We found that the number of MPs in the patients notably increased compared with healthy subjects. However, the number of MPs did not differ in patients with ALI and without ALI. More importantly, the size at peak and interquartile range (IQR) of MPs in patients with ALI are significantly smaller than that of patients without ALI. Our findings suggest that the size distribution of MPs can be used to predict and exclude ALI after cardiac surgery with CPB.

## Materials and Methods

### Patient Selection

The patients who underwent cardiac surgery CPB in the First Affiliated Hospital, Sun Yat-sen University were selected. Inclusion criteria were patients older than 18 years who underwent cardiac surgery with CPB through a mid-sternal incision and with general anesthesia under endotracheal intubation. Exclusion criteria were patients with previous cardiac surgery, previous central nervous system disease, renal failure, hepatic dysfunction, and previous lung disease such as thoracic trauma, pulmonary infection, and chronic pulmonary diseases before operation. The enrolled 103 patients were divided into two groups: patients with ALI (*n* = 25) and patients without ALI (non-ALI, *n* = 78). In addition, 53 healthy subjects were recruited as a control group whose age, gender, as well as other baseline characteristics, were matched with those of the patients included. The study was approved by the Ethics Review Board of the First Affiliated Hospital, Sun Yat-sen University. Prior informed consent was obtained from all subjects who were enrolled in this study.

### Isolation and Size Distribution of MPs

The isolation of MPs followed the methodological guidelines for studying extracellular vesicles (34). All patients and healthy subjects fasted overnight. Blood samples were collected from the peripheral vein about 2.7 ml into a tube with sodium citrate at the time before, 12 h, and 3 d after surgery. The blood samples were centrifuged at 2,000 g for 20 min at 4°C to get platelet-rich plasma. The platelet-rich plasma then was centrifuged at 11,000 g for 2 min at 4°C to obtain platelet-poor plasma. All procedures mentioned above were done 2 h after blood collection. The samples were then stored at −80°C. Platelet-poor plasma was melted at 37°C and then centrifuged at 13,000 g for 45 min to prepare MPs for size distribution detection. MPs were resuspended in phosphate buffer solution to 1.0 ml. The size distribution of MPs was measured by the LitesizerTM 500 Particle Analyzer (Anton Parr, Ashland, VA, United States).

### Nanoparticle Tracking Analysis

The quantification of MPs was determined by the Nanoparticle Tracking Analysis (NTA) system (NS300; Malvern, United Kingdom) as described previously (9). Briefly, the samples were gently inserted into the detection channel followed by setting an ideal resolution and brightness for the observation of MPs' motions. The movement tracks of MPs were recorded for 60 s with a detection threshold optimized for each sample and repeated three times. Data processing was performed by the NTA 3.3 software (Malvern, United Kingdom).

### Data Collection

Data collection included demographics, history, type of cardiac disease; preoperative data such as blood type, blood routine, biochemistry parameter, myocardial enzyme, coagulation, New York Heart Association (NYHA) classification; intraoperative data such as duration of surgery and CPB; postoperative data such as duration of mechanical ventilation, ICU stay, arterial blood gas analysis, and the corresponding data at 12 h and 3 d after surgery when extracting MPs.

### Diagnosis of ALI

The patients after cardiac surgery with CPB were divided into two subgroups (ALI and non-ALI) to further analyze the correlation between size distributions of MPs and acute lung injury. The diagnosis of ALI complies with the standard proposed by the American-European Consensus Conference Committee in 1994 (35): 1. Acute onset. 2. The partial pressure of oxygen /fraction of inspiration O_2_ ≤ 300 mmHg, regardless of positive end-expiratory pressure level. 3. Bilateral infiltrates seen on frontal chest radiograph. 4. < 18 mmHg when measured or no clinical evidence of left atrial hypertension.

### Statistical Analyzes

Statistical analyzes were performed using SPSS 23.0 software (SPSS Inc, Chicago, Ill). For comparison between patients and healthy subjects, an independent 2-sample *t*-test was conducted. To compare the difference before and after surgery (at various time points), repeated measures analyses with Bonferroni Correction were applied. A χ2 analysis was conducted to compare proportions between different groups. The relationship between different indexes of MPs' size distribution was evaluated by Spearman's correlation analysis. Parametric data were presented as mean ± standard deviation (untransformed data) or 95% CI (transformed data). *P* < 0.05 was considered statistically significant.

## Results

### Clinical Characteristics

**Figure 1:**
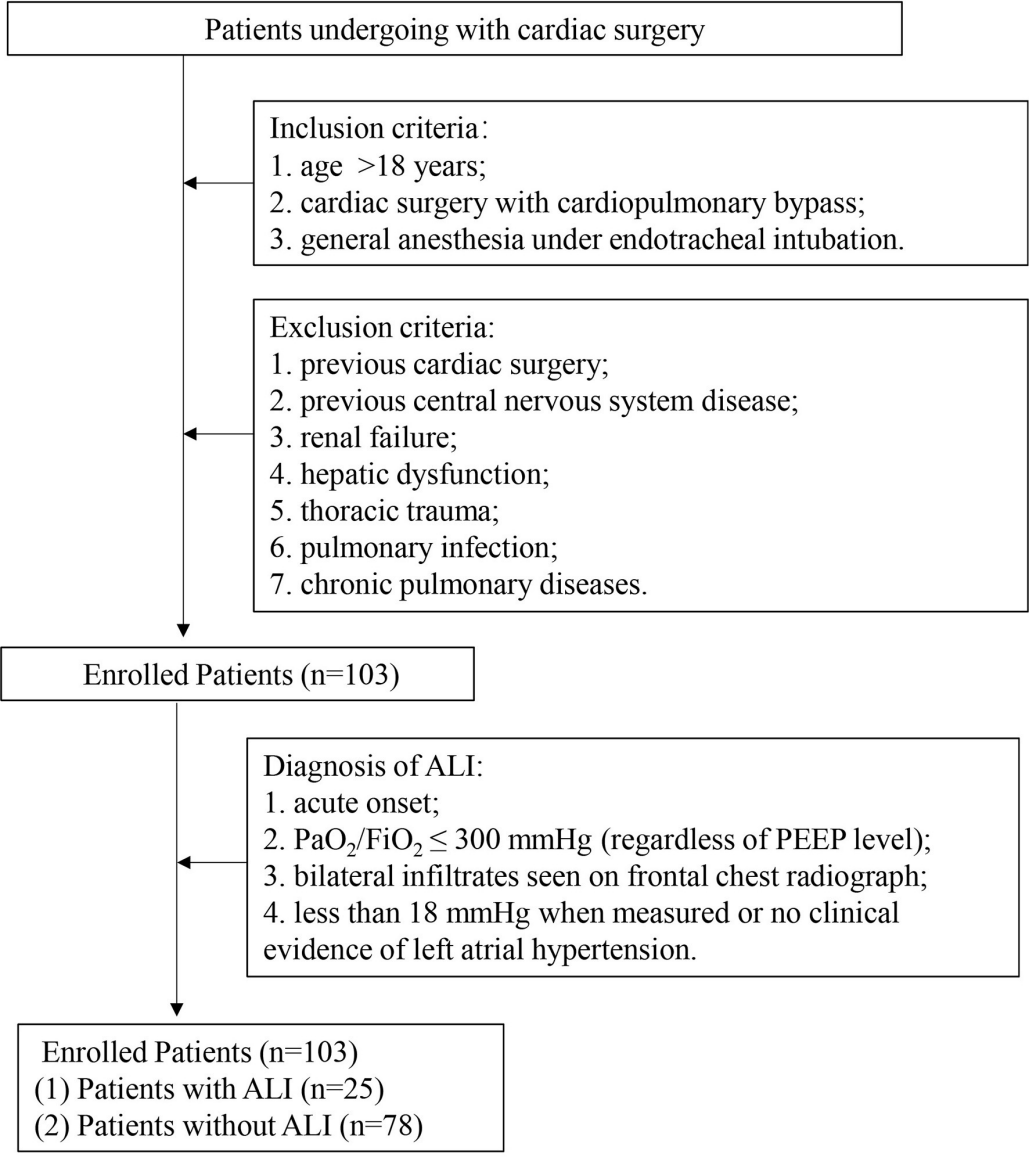
Schematic flow chart of the study design. ALI: acute lung injury; PaO_2_, partial pressure of oxygen; FiO_2_, fraction of inspiration O_2_ and PEEP, positive end-expiratory pressure.

A total of 103 patients were enrolled and 25 (24.3%) of them developed ALI after cardiac surgery (Figure 1). The demographic data and basic perioperative parameters are shown in Table 1. There were no significant differences between the ALI and non-ALI groups in demographic and perioperative characteristics except age (*P* = 0.023). The ALI group tended to have longer CPB time, clamping time, and operation time. The operation data are shown in Table 2.

**Table 1:** Perioperative clinical parameters between the ALI and non-ALI groups.

	**ALI (*n* = 25)**	**non-ALI (*n* = 78)**	***P***
Age (yr)	59.16 ± 12.67	54.57 ± 10.80	0.023*
Female (%)	48.00	30.77	0.874
BMI (kg/m^2^)	25.82 ± 5.49	23.85 ± 3.43	0.107
Drinking (%)	12.00	10.25	0.951
Smoking (%)	20.00	28.21	0.422
Hypertension (%)	40.00	26.92	0.215
Diabetes (%)	12.00	5.13	0.368
Hyperlipidemia (%)	28.00	28.21	0.185
Atrial fibrillation (%)	16.00	23.08	0.457
Coronary disease (%)	52.00	33.33	0.096
Previous myocardial infarction (%)	4.00	1.28	0.396
NYHA classification (I/II/III/IV, n)	1/14/9/1	6/40/28/4	0.922
LVEF (%)	64.12 ± 13.45	63.03 ± 12.92	0.716
CPB time (min)	209.32 ± 106.53	163.88 ± 77.73	0.061
Clamp time (min)	117.48 ± 59.00	95.51 ± 51.83	0.074
Operation time (min)	424.88 ± 171.16	357.22 ± 133.34	0.079

*Data are presented as mean ± standard deviation or number of patients (n, %). ALI, acute lung injury; BMI, body mass index; NYHA, New York Heart Association; LVEF, left ventricular ejection fraction; CPB, cardiopulmonary bypass*.

**Table 2:** The operation data between the ALI and non-ALI groups.

**Surgery**	**ALI (*n* = 25)**	**non-ALI (*n* = 78)**	***P***
Valve	10	43	0.188
CABG	4	9	0.811
Congenital	1	3	1.000
Great artery	2	5	1.000
Valve + CABG	3	5	0.632
Valve + Congenital	1	4	1.000
Others	4	9	0.811

*Data are presented as number of patients (n). ALI, acute lung injury; CABG, coronary artery bypass grafting*.

### Concentration of Circulating MPs in Patients and Healthy Subjects

**Figure 2:**
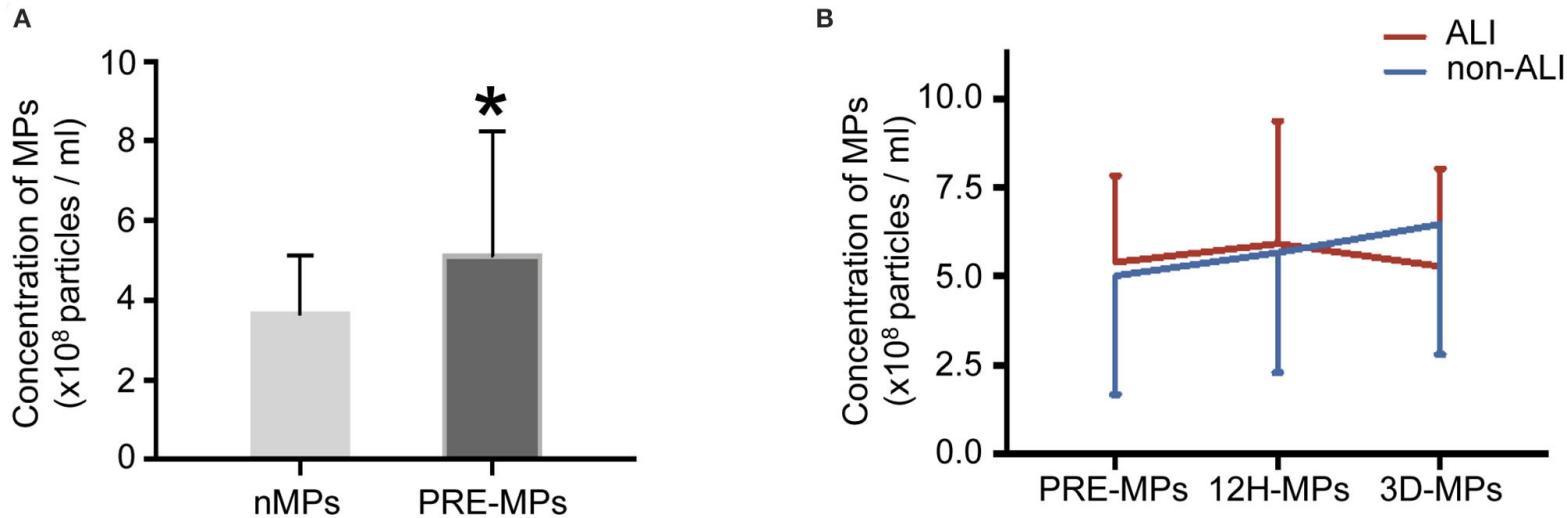
Concentrations of circulating microparticles (MPs) in all patients and healthy subjects. **(A)** The concentrations of MPs in healthy subjects and patients. **(B)** The concentrations of MPs at different perioperative times between ALI and non-ALI groups. ^*^, vs. nMPs, *P* < 0.05. nMPs, MPs from healthy subjects; PRE-MPs, MPs from pre-operation; 12H-MPs, MPs from 12 h after operation; 3D-MPs, MPs from 3 d after the operation.

The number of MPs in the patients notably increased compared with healthy subjects (Figure 2A). However, the ALI group and non-ALI group did not differ in the number of MPs (Figure 2B).

### Comparison of Post-Operative Oxygenation Index (OI) Between ALI and Non-ALI Groups

**Figure 3:**
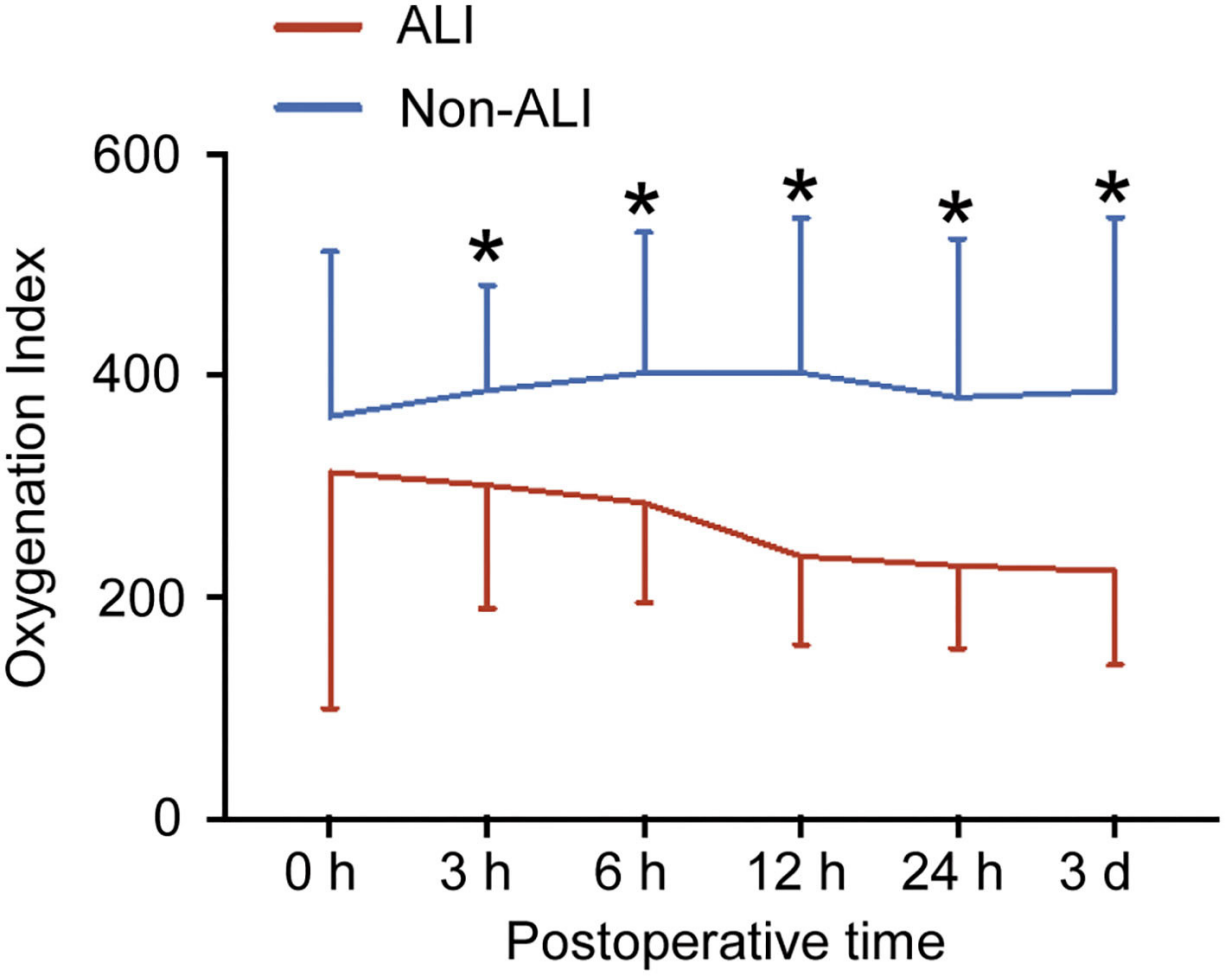
Postoperative oxygenation index (OI) between the ALI and non-ALI groups. The figure showed the OI in patients with and without ALI at different times after surgery. ^*^, vs. ALI group at the same time point, *P* < 0.05.

As shown in Figure 3, OI in the ALI group decreased over time after surgery and reduced to around 240 mmHg at 12 h after surgery. OI in non-ALI patients remained around 390 mmHg. OI between the ALI and non-ALI groups differed significantly at 3 h after operation, and the differences became more pronounced with the extension of time, especially at 12 h after the operation when it showed the steepest drop compared with the non-ALI group. However, OI between 12 h and 3 d after surgery remained steady in the ALI and non-ALI groups. Moreover, OI at 0 h after the operation did not differ between the ALI and non-ALI groups.

### The Size Distribution of MPs in Patients and Healthy Subjects

**Figure 4:**
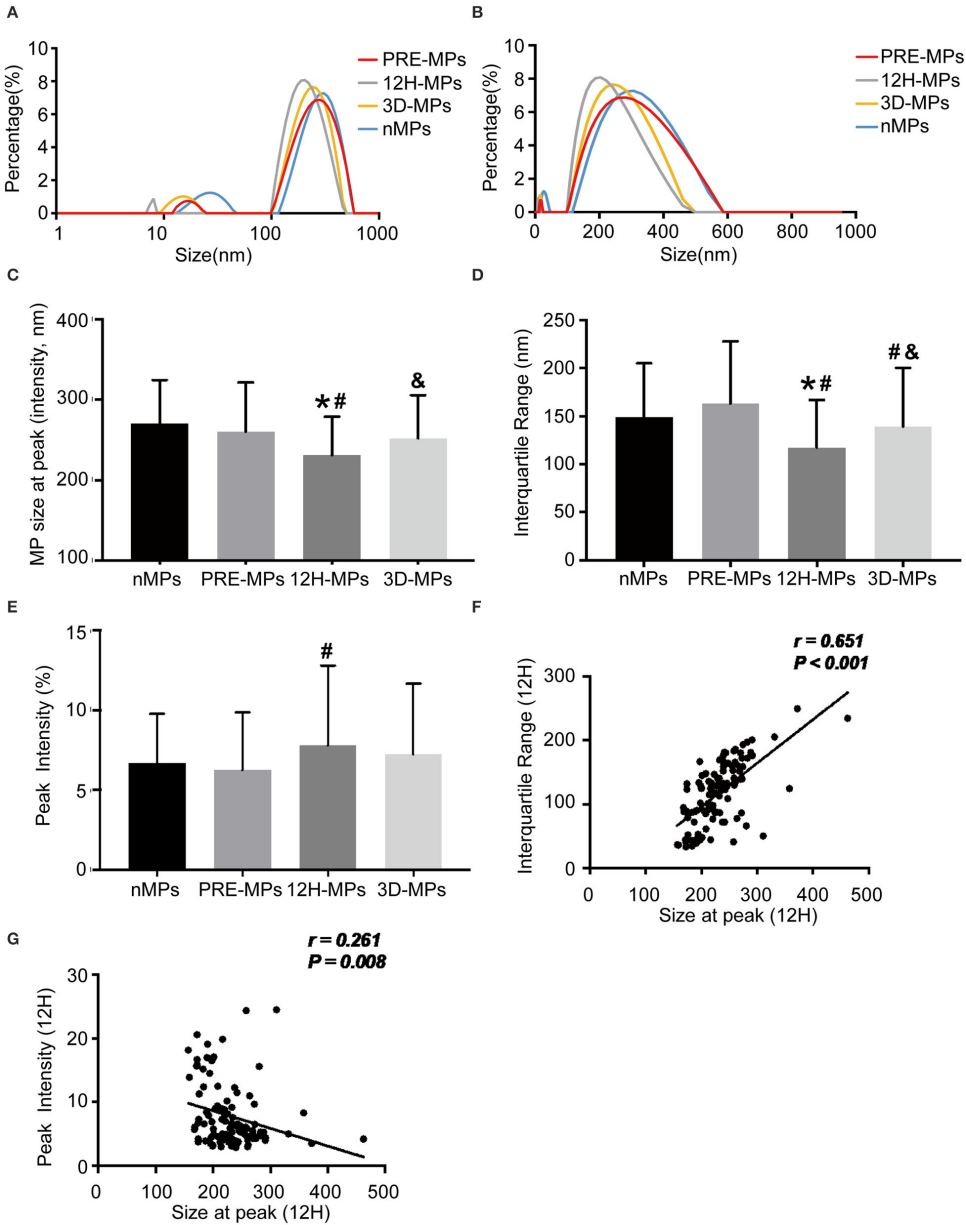
The size distribution of microparticles (MPs) in patients and healthy subjects. **(A,B)**, Representative size distribution curve of MPs in patients **(A,B)** from the same patients using different X-axis. **(C)** The MPs size at peak between patients and healthy subjects. **(D)** The interquartile range (IQR) between patients and healthy subjects. **(E)** The peak intensity between patients and healthy subjects. **(F)** Correlation between size at peak and IQR at 12 h after operation. **(G)** Correlation between size at peak and peak intensity at 12 h after operation. ^*^, vs. nMPs, *P* < 0.05; #, vs. PRE-MPs, *P* < 0.05; &, vs. 12H-MPs, *P* < 0.05. nMPs, MPs from healthy subjects; PRE-MPs, MPs from pre-operation; 12H-MPs, MPs from 12 h after operation; 3D-MPs, MPs from 3 d after operation; 12H, 12 h after operation.

There were two peaks of the size distribution curve of MPs in Figures 4A,B. One was located <100 nm and was considered to be exosomes, and the other was located between 100 and 1,000 nm and was considered to be MPs. The diameters in 80% of the MPs (from the first decile to the last decile) in the patients were between 125 and 280 nm at 12 h after surgery, 150 and 400 nm at pre-operation, and 140 and 360 nm at 3 d after surgery. First, we compared the MPs size distribution of all patients with that of healthy subjects at different time points (Table 3, Figures 4A,B). We found that the MP size at peak of the patients before surgery was slightly < in the healthy subjects (Table 3, Figure 4C). The MP size at peak at 12 h after surgery dramatically decreased to 231.43 nm compared with 260.55 nm at the pre-operation level, which increased to 252.05 nm at 3 d after surgery (Table 3, Figure 4C). The IQR of the MP size in patients before surgery was increased compared with healthy subjects. However, the IQR at 12 h after surgery also dramatically reduced to 117.42 nm compared with 163.29 nm at the pre-operation level, which elevated to 138.98 nm at 3 d after surgery (Table 3, Figure 4D). However, the peak intensity, which means the percentage of MPs at peak, showed no significant difference between healthy subjects and patients before surgery (Table 3, Figure 4E). The peak intensity at 12 h after surgery increased to 7.78% compared with 6.25% in pre-operation, which elevated to 7.24% at 3 d after surgery (Table 3, Figure 4E). The changes in peak intensity are opposite to the changes in IQR after cardiac surgery. In addition, the MP size at the peak was positively correlated with the IQR and negatively correlated with the peak intensity at 12 h after surgery (Figures 4F,G).

**Table 3:** The size distribution of MPs.

	**nMPs**	**PRE-MPs**	**12H-MPs**	**3D-MPs**
Size at peak (nm)	270.92 ± 53.50	260.55 ± 60.88	231.43 ± 47.37^*^^#^	252.05 ± 53.95^&^
Peak intensity (%)	6.69 ± 3.08	6.25 ± 3.61	7.78 ± 5.01^#^	7.24 ± 4.42
IQR (nm)	149.22 ± 55.64	163.29 ± 64.75	117.42 ± 49.49^*^^#^	138.98 ± 61.05^#^^&^

*Data are presented as mean ± standard deviation*.

* *, vs. nMPs, P <0.05;*

# *, vs. PRE-MPs, P <0.05;*

& *, vs. 12H-MPs, P <0.05*.

*nMPs, MPs from healthy subjects; PRE-MPs, MPs from pre-operation; 12H-MPs, MPs from 12 h after operation; 3D-MPs, MPs from 3 d after operation; IQR, interquartile range*.

### The Distribution of MPs Between the ALI and Non-ALI Groups

**Figure 5:**
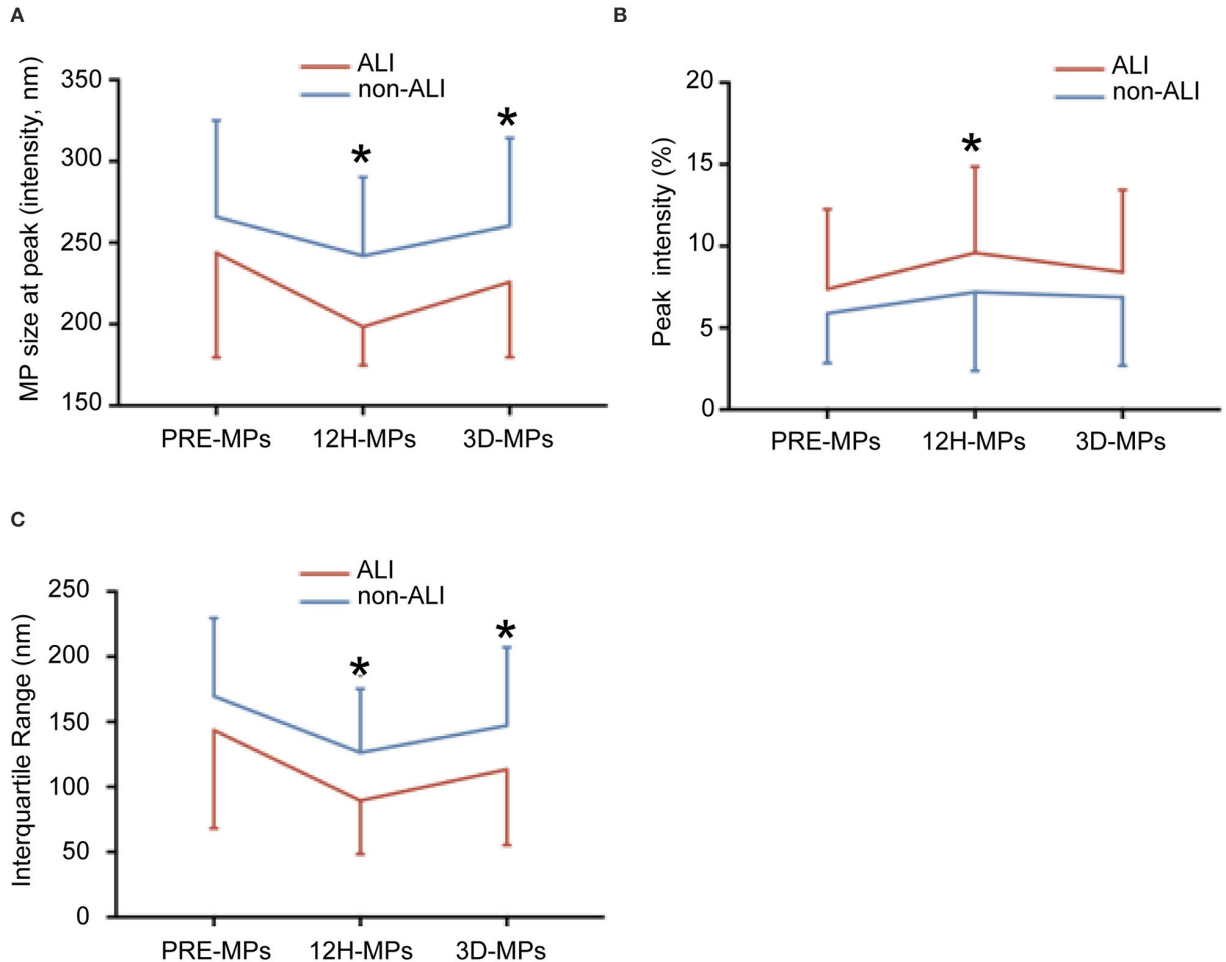
The size distribution of microparticles (MPs) at different times between ALI and non-ALI groups. **(A)** The MPs size at peak between ALI and non-ALI groups. **(B)** The IQR between ALI and non-ALI groups. **(C)** The peak intensity between ALI and non-ALI groups. ^*^, vs. ALI group at the same time point, *P* < 0.05. PRE-MPs, MPs from pre-operation; 12H-MPs, MPs from 12 h after operation; 3D-MPs, MPs from 3 d after the operation.

The perioperative size distribution of MPs is shown in Table 4, Figure 5. Figure 5A, Table 4 show that the size at the peak of MPs in patients with ALI at 12 h and 3 d after surgery were < those of the patients without ALI. The preoperative size at the peak of MPs in patients with ALI was also < those of the patients without ALI, but there was no statistical significance. The peak intensity did not differ between the ALI and non-ALI groups except at 12 h, when the peak intensity in patients with ALI was elevated than those in patients without ALI at 12 h (Figure 5B, Table 4). As for the IQR, there were significant differences between the ALI and non-ALI groups at 12 h and 3 d after surgery, when the values in the patients with ALI were < those in the patients with non-ALI. Meanwhile, there was no statistical significance for the IQR between the ALI and non-ALI groups before surgery (Figure 5C, Table 4).

**Table 4:** Size distribution of MPs between the ALI and non-ALI groups.

**Size distribution of MPs**	**ALI (*n* = 25)**	**non-ALI (*n* = 78)**	***P***
Size at peak (PRE)	243.76 ± 64.15	265.93 ± 59.21	0.114
Peak intensity (PRE)	7.37 ± 4.89	5.89 ± 3.04	0.162
IQR (PRE)	143.57 ± 75.35	169.61 ± 60.14	0.080
Size at peak (12H)	198.49 ± 23.81	241.99 ± 48.27	<0.001
Peak intensity (12H)	9.59 ± 5.27	7.20 ± 4.82	0.037
IQR (12H)	89.40 ± 41.04	126.40 ± 48.82	0.001
Size at peak (3D)	225.84 ± 46.07	260.45 ± 53.84	0.005
Peak intensity (3D)	8.41 ± 5.02	6.87 ± 4.17	0.129
IQR (3D)	113.54 ± 58.15	147.13 ± 60.05	0.016

*Data are presented as mean ± standard deviation. ALI, acute lung injury. PRE, pre-operation; 12H, 12 h after operation; 3D, 3 d after operation; IQR, interquartile range*.

### Correlations Between Perioperative Measurements and ALI

**Figure 6:**
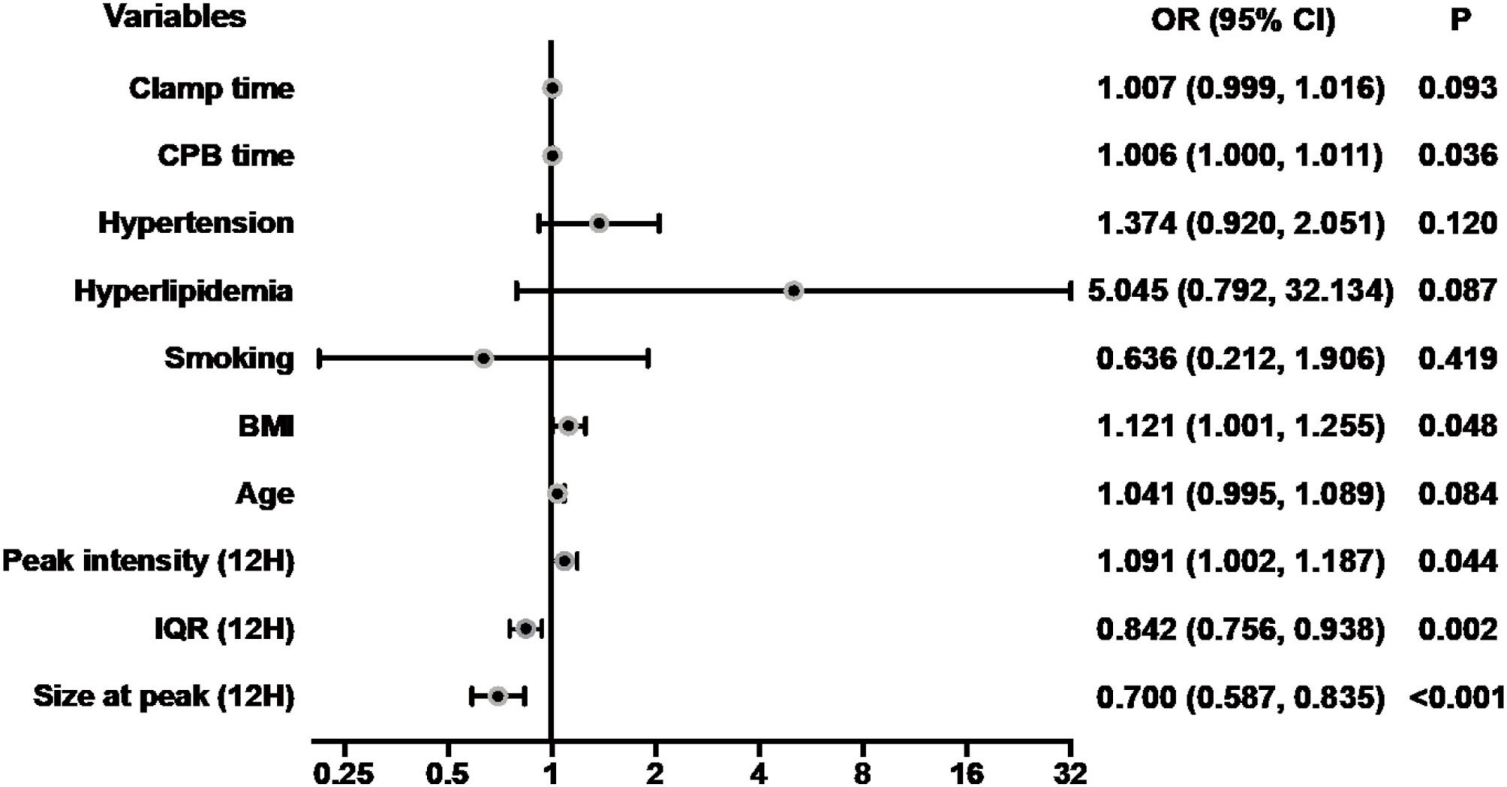
Forest plot for logistic regression analysis. The odds ratios for acute lung injury according to size at peak (12 H) and selected risk factors. CPB, cardiopulmonary bypass; BMI, body mass index; IQR, interquartile range; Size at peak (12H), the size at peak at 12 h after operation; OR, odds ratios; CI, confidence interval.

Since the index of the size distribution of MPs at 12 h after surgery dramatically and repeatedly changes, which might indicate the occurrence of ALI at a very early stage, we further performed the univariate analysis of the size at peak, the IQR, and the peak intensity at 12 h after surgery as well as preoperative clinical parameters potentially associated with ALI as shown in Table 5, Figure 6. There are five main parameters associated closely with ALI: size at peak (12 h), IQR (12 H), peak intensity (12 H), BMI, and CPB time. We put these 5 parameters together to perform a multivariate analysis. The results showed that the size at peak of MPs at post-operative 12 h and CPB time are the independent risk factors for the occurrence of ALI after surgery.

**Table 5:** Univariate and multivariate analyses of ALI.

	**Univariate**		**Multivariate**	
	**OR (95 % CI)**	***P***	**OR (95 % CI)**	***P***
Size at peak (12H) ^a)^	0.700 (0.587, 0.835)	<0.001	0.687 (0.557, 0.846)	<0.001
IQR (12H) ^a^	0.842 (0.756, 0.938)	0.002		
Peak intensity (12H)	1.091 (1.002, 1.187)	0.044		
Age	1.041 (0.995, 1.089)	0.084		
BMI	1.121 (1.001, 1.255)	0.048		
Smoking	0.636 (0.212, 1.906)	0.419		
Hyperlipidemia	5.045 (0.792, 32.134)	0.087		
Hypertension	1.374 (0.920, 2.051)	0.120		
CPB time	1.006 (1.000, 1.011)	0.036	1.008 (1.001, 1.014)	0.015
Clamp time	1.007 (0.999, 1.016)	0.093		

a * Measurement unit: ×10 nm. ALI, acute lung injury; OR, odds ratio; CI, confidence interval; 12H, 12 h after operation; IQR, interquartile range; BMI, body mass index; CPB, cardiopulmonary bypass*.

### Receiver Operating Characteristic Curve Analysis

**Figure 7:**
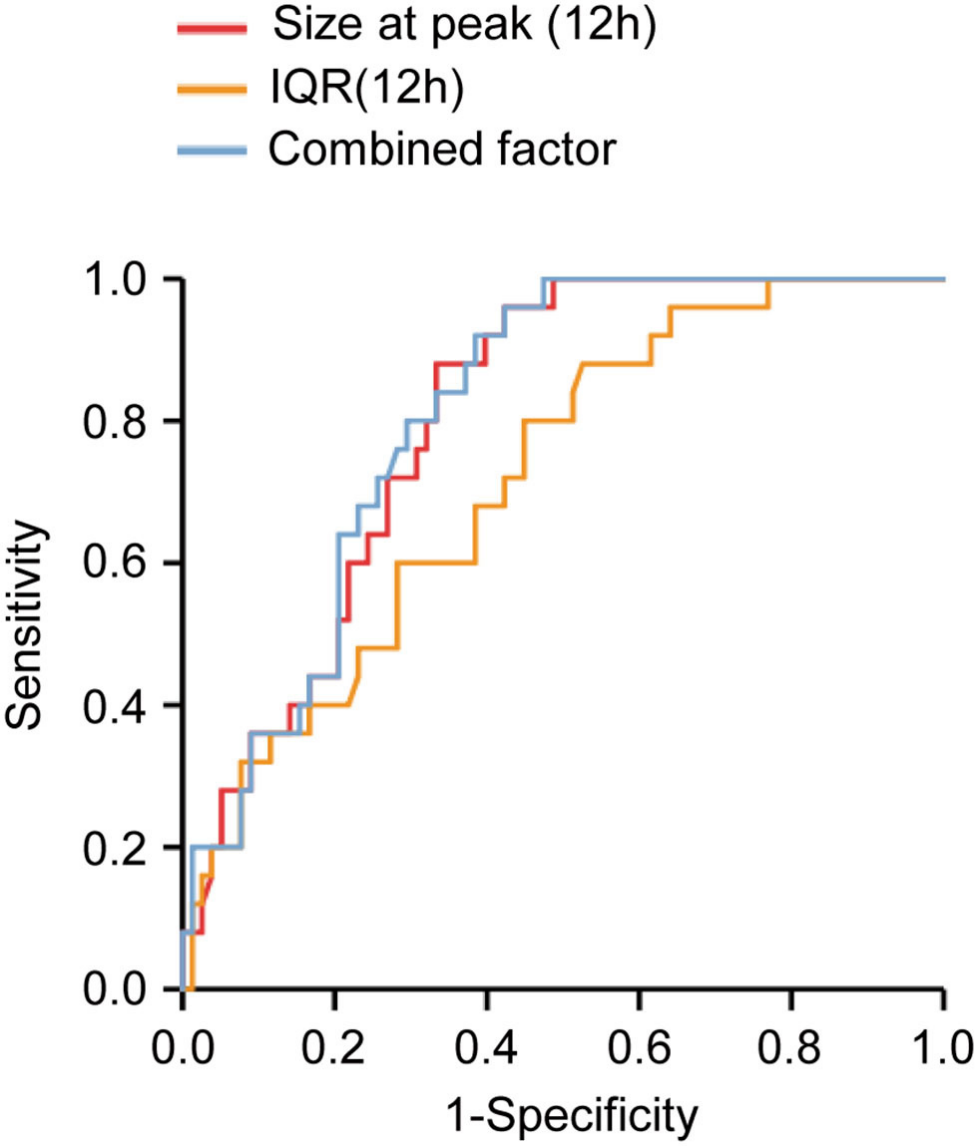
Receiver operating characteristic curve analysis of microparticles (MPs) size distribution determined at 12 h after operation. The areas under the curves were 0.803, 0.717, and 0.809 for size at peak (12H), IQR (12H), and the combined factor, respectively. Size at peak (12H), the size at peak at 12 h after operation; IQR (12H), the IQR at 12 h after operation; combined factor, the combined factor of size at peak (12H) and IQR (12H).

The value of MP size distribution in predicting the occurrence of ALI was further evaluated using the receiver operating characteristic curve (Figure 7). The area under the curve (AUC) of peak diameter at 12 h was 0.803. Table 6 shows that the best cutoff value for the size at peak at 12 h after surgery to diagnose ALI was 223.05 nm with a sensitivity of 88.0% and a specificity of 66.7%. The positive predictive value (PPV) and negative predictive value (NPV) were 0.458 and 0.945, respectively. As for the IQR of 12 h after surgery, the AUC was 0.717. The best cutoff value for the IQR to diagnose and exclude ALI was 132.65 nm with a sensitivity of 88.0% and a specificity of 47.4%. The PPV and NPV are 0.349, and 0.925, respectively. Combining these two parameters (size at peak and IQR) as a combined diagnostic factor for analysis, the AUC was 0.809, the sensitivity went up to 0.92, and the NPV reached 0.96.

**Table 6:** Receiver operating characteristic curve.

	**AUC**	**Cutoff value**	**At cutoff value**			
		**(True positive cases)**	**Sensitivity**	**Specificity**	**NPV**	**PPV**
Size at peak (12H)	0.803	223.050 (22) ^a^	0.880	0.667	0.945	0.458
		194.745 (10) ^b^	0.400	0.846	0.815	0.455
		239.200 (25) ^c^	1.000	0.513	1.000	0.397
IQR (12H)	0.717	132.650 (22) ^a^	0.880	0.474	0.925	0.349
		69.200 (9) ^b^	0.360	0.872	0.810	0.474
		166.700 (25) ^c^	1.000	0.231	1.000	0.294
Combined factor ^d^	0.809	241.532 (23) ^a^	0.920	0.615	0.960	0.434
		201.078 (8) ^b^	0.400	0.846	0.790	0.364
		251.873 (25) ^c^	1.000	0.526	1.000	0.403

a * Best cutoff value*.

b * Cutoff value with specificity about 85%*.

c * Cutoff value with sensitivity up to 100%*.

d * The combined factor of size at peak (12H) and IQR (12H)*.

*AUC, area under the curve; NPV, negative predictive value; PPV, positive predictive value; 12H, 12 h after operation; IQR, interquartile range*.

When the size at peak at 12 h after surgery reached 239.2 nm, the sensitivity was 100% with a specificity of 51.3% and 1 of NPV, whereas when the size at peak at 12 h after surgery reached 194.745 nm, the specificity was 84.6% with a sensitivity of 40% and 0.455 of PPV. When the IQR at 12 h after surgery increased to 166.7 nm, the sensitivity was 100% with a specificity of 23.1% and 1 of NPV, whereas when the IQR at 12 h after surgery decreased to 69.2 nm, the specificity was 87.2% with a sensitivity of 36% and 0.474 of PPV. Combining these two parameters as a combined diagnostic factor, we found that when the value was 251.873 nm, the sensitivity was 100% with a specificity of 52.6% and 1 of NPV, whereas when the value was 201.078 nm, the specificity was 84.6% with a sensitivity of 40% and 0.364 of PPV (Table 6).

## Discussion

ALI is a severe complication after cardiac surgery with CPB and the incidence is up to 60%. Unfortunately, there is no parameter available to predict or exclude ALI after cardiac surgery with CPB. In the past decade, we and other researchers focused on the role of MPs in ALI, the different origins of MPs (endothelial microparticles, monocyte microparticles, platelet-derived microparticles, leukocyte microparticles), especially the concentration, types, and compositions of MPs, and demonstrated that MPs may participate in the development of ALI (11, 12, 16–18, 28, 30, 32, 33, 36–40). Thus, MPs may be a diagnostic marker and treating target in the early stage of ALI. In this study, we found that the size distribution of MPs can be used to predict and exclude ALI after cardiac surgery with CPB.

MPs have been described as cell-derived membrane vesicles of 100–1,000 nm diameter containing proteins, DNAs, and cytosolic materials in extracellular spaces (41). MPs were analyzed with a conventional flow cytometer in many studies. Previously, we found the endothelial microparticles detected by the flow cytometer are increased after cardiac surgery (10). The levels of endothelial microparticles seem to have no correlation with acute lung injury after cardiac surgery probably as a result of inadequate detection for microparticles with diameters < 300 nm. Because of the inherent limitations of the conventional flow cytometer, as these instruments were traditionally developed to measure whole cells, which are orders of magnitude larger and express far more molecules of identifying epitopes, this approach cannot accurately measure the MPs smaller than 300 nm in diameter. Although electron microscopy and atomic force microscopy can detect the MPs with a diameter <300 nm (42), it is unpractical in clinical measurement since it is time-consuming. Some studies used the conventional flow cytometry plus polystyrene beads of 110, 200, 500 nm, and 1 mm diameter to set up the MP size gate in two small angle light scatter detectors to analyze MPs smaller than 300 nm in diameter. However, it was difficult to separate the background noise with the MPs smaller than 300 nm and effectively measure the size of MPs by different diameter groups, so it was not able to precisely calculate the size of each MP (43, 44). Recently, NTA has been applied to mathematically calculate the concentration and size distribution of MPs, which records the path of each particle to determine the mean velocity and diffusivity, since the particles undergo Brownian motion (45). We adopted this approach to analyze the concentration of MPs in the present study. However, for accurate measurement of the size distribution of MPs, the NTA procedure requires accurate optimization of camera and analysis settings. Separate detections with different settings may be needed to obtain accurate readings for MPs subsets in heterogeneous mixtures (45). Therefore, in this study, we used a LitesizerTM 500 to measure the size distribution of MPs by dynamic light scattering, a technique to calculate the characteristic time of the fluctuations originating from the Brownian motion of MPs in scattered light intensity, and obtained the results using the Einstein-Stokes equation (46–54). This technique can detect the average particle diameter and particlediameter distribution of nano-sized particles dispersed in a liquid. Using this technique, we found that the diameters in most of the MPs were around 170–300 nm at 12 h after surgery, 175–500 nm before surgery, and 175–500 nm at 3 d after surgery, indicating that using a conventional flow cytometer to measure MPs can only detect a small part of MPs previously, and most of the MPs cannot be detected, especially at 12 h after surgery.

Using this technique, we found that the size of MPs is smaller in patients than in healthy subjects. More importantly, the size of MPs is dramatically reduced after cardiac surgery with CPB, especially at postoperative 12 h. The same happened in the IQR, which is smaller after surgery, especially at postoperative 12 h. On the contrary, the peak intensity increases after the surgery, especially at postoperative 12 h. The curve of the size distribution of MPs seems to be able to shift from right to left (becomes small) with the shape becoming high and narrow (sharp shape) after cardiac surgery with CPB, especially at postoperative 12 h. Why the size distribution of MPs after cardiac surgery is altered remains unclear. One speculation is larger MPs may be filtered by the cardiopulmonary bypass membrane during cardiac surgery. There are a few reports on the changes in sizes or diameters of circulating microparticles under pathological conditions (55–57). The circulating extracellular vesicles (also called microparticles) derived from the ALI patients showed smaller sizes compared with control healthy subjects, but with no significant difference, which may be the result of the insufficiency of enrolled patients (57). The diameters of circulating microparticles isolated from the patients with chronic fatigue syndrome were significantly smaller than those derived from non-fatigued healthy controls (55). In the pig model of the metabolic syndrome, the mesenchymal stromal cells-derived extracellular vesicles were on average smaller in size compared with the extracellular vesicles from the pig control (56). The sizes of circulating MPs are altered under different pathological states and the correlation between the changes and ALI after cardiac surgery is unclear. When further comparing the peak diameter and the IQR of MPs at 12 h after surgery, we found that the peak diameter and the IQR of MPs decreased more significantly in patients with ALI than in patients without ALI, suggesting that the MPs' generation is different before and after the operation, and between the ALI and non-ALI groups. It seems that cardiac surgery with CPB and ALI can stimulate the production of small-sized MPs, which may be used as a biomarker. Indeed, it has been reported that there were increased levels of small-sized MPs in patients with psoriasis (58), which is consistent with our findings. Thus, small-sized MPs may be generated under disaster stress and numerous inflammations, and the true mechanisms need to be further investigated in the future. Another finding in this study is the size distribution of MPs also shifts from right to left (being smaller) with the shape becoming high and narrow (sharp shape) before surgery in patients with ALI compared to the non-ALI group, although there is no statistical significance (may get statistically significant when increasing the sample size), indicating that patients with the small-size and sharp shape distribution of MPs are prone to ALI after surgery.

As mentioned above, the peak diameter and the IQR of MPs at 12 h after surgery changed most significantly. We further analyzed the relationship between these parameters and ALI. We found a certain relationship between peak diameter at 12 h after surgery and ALI in the present study (AUC = 0.803). Although the diagnostic specificity of peak diameter at the best cutoff value is about 66.7%, the diagnostic sensitivity is very high, which reached 88% with a 0.945 negative predictive value. More importantly, the peak diameter of MPs can be used as an exclusive index of ALI when the sensitivity reaches 100%, and the peak diameter at 12 h after surgery is 239.2 nm. In other words, the diagnosis of ALI could be excluded if the peak diameter at 12 h after surgery is larger than 239.2 nm. We can also increase the diagnostic specificity to 84.6% with a negative predictive value of 0.815 by setting a peak diameter smaller than 194.745 nm. Moreover, the IQR of postoperative 12 h also has a certain value in the prediction of ALI. Combining peak diameter and IQR through regression equation at the best cutoff value, the diagnostic sensitivity can reach 92% with a negative predictive value of 0.96. We can also set a certain value of IQR and the combined peak diameter and IQR at 12 h after surgery to reach 100% of sensitivity and more than 85% of specificity to exclude and diagnose ALI. The question is how to diagnose ALI if the peak diameter is between 194.745 and 239.2 nm, the IQR is between 69.2 and 166.7 nm, or the combined factor is between 201.078 and 251.873 nm, which accounts for about 40% of the patients. In this situation, we can combine clinical manifestations and other diagnostic methods to predict ALI. Indeed, further logistic regression analyses suggested that the size of MPs at postoperative 12 h was an independent risk factor for the occurrence of ALI. This is very important since ALI is a severe complication with a great impact on cardiopulmonary function, and postoperative 12 h is a very early stage after cardiac surgery, and doctors can distinguish suspicious ALI patients and conduct further inspection and intervention to prevent the occurrence of ALI or treat patients at an earlier stage of ALI. Therefore, it is of great value to use the size distribution of MPs to predict and exclude ALI after cardiac surgery with CPB.

In this study, the OI of the two groups did not significantly differ at postoperative 0 h. However, the OI of ALI patients decreased gradually with time after surgery. It seemed that the pathological changes of the lung had appeared in the early period after the operation, and earlier than the measurement indexes detected by existing methods, such as artery blood gas analysis and chest radiography. The OI of ALI patients decreased apparently in the first 12 h, and then remained stable until 3 d. This is also the reason the three time points were chosen (pre, 12 h, 3 d) in this study.

Limitation: 1. This is a single-center study and the sample size of 103 patients is relatively small. Multicenter researches and an enlarged sample are needed in future studies. 2. The preoperative OI of patients was not obtained, since arterial blood gas analysis was not performed regularly before the operation. If there was no significant difference in preoperative OI between the ALI and non-ALI groups, MPs will be of higher value in predicting postoperative ALI. 3. We chose three time points (pre, 12 h, 3 d) in this study. If we can monitor frequently or even dynamically, the relationship between ALI and the size distribution of MPs will be more explicit.

Conclusion: Our data showed the peak diameter and the IQR of MPs at postoperative 12 h are smaller in patients with ALI than those in the non-ALI group after cardiac surgery with CPB. This is the first demonstration of the relationship between the size distribution of MPs and ALI after cardiac surgery with CPB. Our findings suggested that the size distribution of MPs could be a novel biomarker to predict and exclude ALI after cardiac surgery with CPB.

## Data Availability Statement

The raw data supporting the conclusions of this article will be made available by the authors, without undue reservation.

## Ethics Statement

The studies involving human participants were reviewed and approved by Ethics Review Board of the First Affiliated Hospital, Sun Yat-sen University. The patients/participants provided their written informed consent to participate in this study.

## Author Contributions

J-SO, YL, and Z-JO: conception and design. H-XY, CC, Y-QL, X-JL, Y-TC, K-FL, Y-PJ, J-SL, and Y-QX: acquisition of data. CC and Y-QL: analysis and interpretation of data. J-SO, YL, Z-JO, H-XY, K-FL, CC, and Y-QL: drafting or revising of the article. All authors contributed to the article and approved the submitted version.

## Funding

This work was supported by the National Key R&D Program of China (2021YFA0805100), the National Natural Science Foundation of China (81830013, 81970363, 81770241, and 82000362), Guangdong Basic and Applied Basic Research Foundation (2019B1515120092), Science and Technology Planning Project of Guangzhou, China (202103000016), the Sun Yat-sen University Clinical Research 5010 Program (2014002), and Program of National Key Clinical Specialties.

## Conflict of Interest

The authors declare that the research was conducted in the absence of any commercial or financial relationships that could be construed as a potential conflict of interest.

## Publisher's Note

All claims expressed in this article are solely those of the authors and do not necessarily represent those of their affiliated organizations, or those of the publisher, the editors and the reviewers. Any product that may be evaluated in this article, or claim that may be made by its manufacturer, is not guaranteed or endorsed by the publisher.

## Abbreviations

ALI: Acute lung injury
CPB: Cardiopulmonary bypass
MPs: Microparticles
AUC: Area under the curve
IQR: Interquartile range
NTA: Nanoparticle Tracking Analysis
NYHA: New York Heart Association
OI: Oxygenation index
PPV: Positive predictive value
NPV: Negative predictive value
DLS: Dynamic light scattering.
